# Detection of a Thermal Stable-Soluble Protein (TSSP) as a Marker of Peanut Adulteration Using a Highly Sensitive Indirect Enzyme-Linked Immunosorbent Assay based on Monoclonal Antibodies

**DOI:** 10.4014/jmb.2304.04038

**Published:** 2023-05-30

**Authors:** Sol-A Kim, Sazzad Hossen Toushik, Jeong-Eun Lee, Won-Bo Shim

**Affiliations:** 1 Division of Applied Life Science, Graduate School, Gyeongsang National University, Jinju, Gyeongnam 52828, Republic of Korea; 2 Department of Microbiology, Stamford University Bangladesh, Dhaka 1217, Bangladesh; 3 Institute of Smart Farm Research Center, Gyeongsang National University, Jinju 52828, Republic of Korea; 4 Institute of Agricultural and Life Science, Gyeongsang National University, Jinju 52828, Republic of Korea; 5 Division of Food Science and Technology, Gyeongsang National University, Jinju 52828, Republic of Korea

**Keywords:** Peanut, thermal stable-soluble proteins (TSSPs), monoclonal antibody, food allergy, ELISA

## Abstract

Food allergy represents a severe problem for many societies, including sensitive populations, academies, health authorities, and the food industry. Peanut allergy occupies a special place in the food allergy spectrum. To prevent consumption by consumers suffering from a peanut allergy, a rapid and sensitive detection method is essential to identify unintended peanut adulteration in processed foods. In this study, we produced four monoclonal antibodies (MAbs; RO 3A1-12, PB 4C12-10, PB 5F9-23, and PB 6G4-30) specific to thermo-stable and soluble proteins (TSSPs) of peanut and developed an enzyme-linked immunosorbent assay (ELISA) based on the MAbs. Among them, PB 5F9-23 MAb was firmly bound to Ara h 1, and other MAbs strongly reacted to Ara h 3 in the Western blot analysis. An antibody cocktail solution of the MAbs was used to enhance the sensitivity of an indirect ELISA, and the limit of detection of the indirect ELISA based on the antibody cocktail solution was 1 ng/ml and improved compared to the indirect ELISA based on the single MAb (11 ng/ml). The cross-reaction analysis revealed the high specificity of developed MAbs to peanut TSSPs without cross-reaction to other food allergens, including nuts. Subsequently, analyzing processed foods by indirect ELISA, all foods labeled as containing peanuts in the product description were confirmed to be positive. The results indicate that the developed antibodies exhibit high specificity and sensitivity to peanuts and can be used as bio-receptors in immunoassays or biosensors to detect intentional or unintentional adulteration of peanuts in processed foods, particularly heat-processed foods.

## Introduction

Food allergies cause abnormal immune reactions with symptoms that can target organs throughout the body [1], including the derma, digestive, respiratory, cardiovascular, and neurological systems [2]. Recent years have witnessed an upsurge in food allergy cases across most countries [3]. Developed countries are primarily affected by food allergy-associated problems, but those incidents have also increased rapidly in developing countries in proportion to economic growth [4]. Peanut is one of the top 9 food allergens (sesame has been newly classified since January 2023), and people with peanut allergies often experience severe allergic reactions in the USA [5]. Recently, 9 pesto products have been recalled because they contained undeclared peanuts in Australia (2020), and the intentional and unintentional adulteration of peanuts has often been reported in nut and sauce products [6].

Since food allergens cannot be recognized with the naked eye in processed foods, the best way to protect people suffering from food allergies is to avoid consuming the allergens that trigger them [7]. Therefore, many countries have mandated labeling on processed foods and foods served in restaurants if they contain food allergens. However, food allergy-associated accidents are still increasing due to insufficient food allergen control in complex food processing facilities and intentional adulteration by food fraud for economic reasons [8]. Moreover, allergy sufferers can be significantly affected in the long-lasting by consuming undeclared food allergens in foods [6]. Consequently, there is an urgent need to develop analytical methods for the easy, sensitive, and specific detection of food allergens and adulterants in processed foods.

Molecular diagnostic techniques, such as conventional and real-time PCR, amplify and analyze specific DNA sequences, and they are frequently used in various fields, including disease diagnosis [9]. However, molecular diagnostic methods have limitations because they require complex analysis processes, expensive equipment, and a trained person [10]. Mass spectrometry based on molecular weight information has often been used to identify proteins or peptides produced, but its quantification is challenging. Unfortunately, protein identification in complex mixtures is difficult and requires expensive equipment and additional preparation time, making it unsuitable for analyzing large numbers of samples [11]. Immunoassays based on specific antibodies can detect low concentrations of analytes in complex samples with minimal sample preparation and extraction. When determining food allergens in food facilities, immunoassays targeting specific proteins are the obvious choice [12, 13]. Moreover, enzyme-linked immunosorbent assays (ELISAs) and lateral flow assays have been well-established and used in food allergen analysis as the gold standard [14].

Various commercialized ELISA kits can specifically analyze peanut proteins. The change of structural and chemical properties of natural peanut proteins due to common heat treatments used in the food industry, such as cooking, sterilization, and autoclaving, should be worth noting as it can result in the loss of their antigenicity [15]. Therefore, developing immunoassays to determine peanut allergens in processed foods is required to use thermal stable-soluble proteins as biomarkers that maintain antigenicity even after the heat treatment. Thus, this study aimed to investigate the presence of thermal stable-soluble proteins (TSSPs) in roasted peanut and peanut butter and its potential as an immunogen to develop sensitive monoclonal antibodies for rapid and straightforward detection of peanuts in processed foods.

## Materials and Methods

### Chemicals and Equipment

0.05 M phosphate-buffered saline (PBS; pH 7.4), 0.5 M sodium chloride (NaCl; pH 6.5), 0.02 M Tris-hydrochloride (Tris-HCl; pH 7.4), 0.1 M carbonate buffer (pH 9.6), and 0.025 M Tris-buffered saline (TBS; pH 7.4) from Sigma-Aldrich Co. (USA) were used as extraction buffers to extract TSSPs from peanut. Natural peanut (*Arachis hypogaea*) proteins Ara h 1 (64.5 kDa, monomer), h 2 (17-19 kDa, doublet), and h 3(14-45 kDa, Multiple subunits) were obtained from Indoor Biotechnologies Ltd. (UK). For sodium dodecyl sulfate-polyacrylamide gel electrophoresis (SDS-PAGE) and Western blot analysis, the Bradford (Quick Start Bradford protein assay), AP conjugate substrate kit, goat anti-mouse IgG-AP conjugate, acrylamide/bis-acrylamide, 30% solution (37.5:1), Precision Plus Protein Kaleidoscope prestained protein standards (10–250 kDa), PowerPac basic power supply, and Mini-PROTEAN tetra cell were purchased from Bio-Rad (USA). 10 × Tris/Glycine/SDS and the polyvinylidene difluoride (PVDF) membrane were obtained from AccuGENE (Korea) and Millipore (USA), respectively.

For the culture of hybridoma cells, T-25 and T-75 flasks, cryogenic vials, 15 ml and 50 ml conical tubes, and cell culture plates (96-well) were purchased from SPL Life Sciences Co., Ltd. (Korea). Roswell park memorial institute (RPMI) 1640, Dulbecco’s modified eagle’s medium (DMEM), and Fetal bovine serum (FBS) from Hyclone (USA) were obtained. To develop an indirect ELISA, goat anti-mouse IgG (H+L) peroxidase conjugate and 96-well Microtiter microplate were obtained from Thermo Fisher Scientific, Inc. (USA). EZblue gel staining reagent, 2,2'-azino-bis-(3-ethylbenzothiazoline-6-sulfonic acid) (ABTS) solution, and bovine serum albumin (BSA) were purchased from Sigma-Aldrich Co. The 8-channel microplate washer (HydroFlex) and a multimode microplate reader (Spark 10M) were obtained from Tecan Trading AG (Switzerland) for the spectrophotometric investigation in this study.

### Preparation of Peanuts and Other Food Samples

Peanut buffer (Skippy^®^, Hormel Food, USA) and raw peanuts were purchased from local markets (Korea) and used to extract TSSPs, which could be used as immunogens and coating antigens in further experiments. Steamed peanuts were obtained by subjecting them to steam heating at a temperature of 100°C for a duration of 30 min, while the roasting process involved heating the peanuts in a roaster (HT-2,000; Henz, Korea) at 150°C for 30 min. Almond, cashew nut, soybean, wheat, sesame, shrimp, mackerel, pork meat, egg white, and milk samples were also purchased from the local market and used to investigate the specificity of the monoclonal antibodies (MAbs) developed in this study.

### The Extraction of TSSPs from Peanuts

TSSPs in peanuts were extracted by the extraction method with slight modifications reported by Kim *et al*. [16]. Peanut butter, raw, and processed peanuts (steamed and roasted) were used to investigate the presence of TSSPs. One gram of each sample was mixed with 10 ml of carbonate buffer (0.1 M, pH 9.6). The extraction of soluble protein was carried out by maintaining for 1 h at 4°C (non-heating extraction) or heating the samples to boiling water for 15 min (heating extraction). The liquid part was transferred into 50 ml conical tubes, centrifuged at 3,220 ×*g* for 15 min at 4°C, and filtered through Whatman No. 4 filter paper (UK). According to the manufacturer’s instructions, the protein concentration of the extracts was determined using Quick start Bradford protein assay dye. Additionally, samples for investigating cross-reactivity, changes in antigenicity, and analysis of processed foods were extracted by the heating extraction method (100°C, 15 min). The soluble protein composition in the extracts was analyzed via the SDS-PAGE, and Ara h 1, 2, and 3 were used as reference proteins.

Five buffers (0.05 M PBS, 0.1 M carbonate buffer, 0.5 M NaCl, 0.025 M TBS, and 0.02 M Tris-HCl) were tested as an extraction buffer to determine the TSSPs extraction efficiency from processed peanuts (steamed, and roasted peanut) and peanut butter using the heating extraction method. The extraction efficiency was also evaluated by measuring the concentration of total soluble protein.

### Production of MAbs against TSSPs of Peanut

TSSPs from roasted peanut and peanut butter by the heating extraction method were respectively used as immunogens. Extraction and filtration steps were performed in the same manner as described above, and the filtrates were dialyzed with 0.05 M PBS (pH 7.4) for 3 days.

Compliance with the institutional animal care and use committee (IACUC) practices at Gyeongsang National University (Korea) was ensured during the handling of all animals (GNU-221103-M0153-01). Six female BALB/c mice (6-week old) were immunized with crude TSSP extracts. For the first boosting, 100 μl of each TSSP solution (1 mg/ml) was emulsified with 100 μl of Freund's complete adjuvant and injected into the peritoneal cavity of each mouse. Next, the crude TSSPs extracts were emulsified using 100 μl of Freund's incomplete adjuvant, followed by a second and third boosting at 2-week intervals. Three days after the third injection, tail vein sera from mice were collected, and titers were checked via indirect ELISA. In final boosting, 200 μl of immunogen was injected into the mouse peritoneal cavity 3 days before the cell fusion. The development of hybridoma cells producing MAbs was performed according to the standard protocols [17, 18]. Cloned hybridomas producing MAbs specific for peanut proteins were screened by indirect ELISA. For mass-producing MAbs, the ascites fluid was gathered from the mice that received the hybridomas at 1 × 10^7^ cells and purified using ammonium sulfate saturation (60%). The purified MAbs were then lyophilized and stored at −20°C for further investigations.

### Characterization of MAbs Specific to Peanut TSSPs

Cross-reactivity, the presence of TSSPs in peanuts, and the specificity of MAbs were evaluated through Western blot and indirect ELISA analyses [19]. Original extracts were utilized for indirect ELISA, while extracts adjusted to 3 μg of total soluble protein were used for SDS-PAGE, with Ara h 1, 2, and 3 proteins used as reference proteins.

Firstly, the extracts and reference proteins were analyzed via the slightly modified SDS-PAGE (12% gel) method [20]. Briefly, samples (3 μg protein per well on stacking gel) were loaded and separated at 120 V for 2 h. One of the two gels was subjected to staining and de-staining steps, and the other was used for Western blot analysis with a slightly modified method [21]. Proteins in the gel were transferred to a PVDF membrane at 350 mA for 1 h. Afterward, the membrane was blocked overnight at 4°C using TBS containing 3% skim milk (skim milk-TBS), followed by 3 washes with TBS containing 0.05% (v/v) Tween-20 (TBST). The washed membrane was immersed in the culture supernatants (1:2 in skim milk-TBS) or diluted MAbs (1:10,000 in skim milk-TBS), incubated on a rocker at 37°C for 2 h, and the surfeit MAbs were washed 4 times by TBST. Membranes were blocked and incubated with goat anti-mouse IgG-AP conjugate in skim milk-TBS (1:3,000) at 37°C for 1 h and then washed 5 times with TBST. The membrane color development was performed in addition to the AP conjugate substrate kit and removed using the TBST, before further being washed with distilled water. A purplish band appeared visually on the Western blot where the MAb reacted to the TSSPs. The developed blot on the membrane was visualized using Fusion FX (Vilber Lourmat, Germany).

### Optimization of Indirect ELISA for Peanut Detection

An indirect ELISA was optimized by determining several crucial experimental factors including coating, blocking, and primary antibody steps for the rapid and sensitive detection of peanuts in processed foods. The optimized indirect ELISA procedures are as follows: the 96-wells of the microplate were coated with 100 μl of sample extracts tested in 0.1 M carbonate buffer and incubated at 37°C for 1 h. After 3 times washing with PBS containing 0.05% (v/v) Tween-20 (PBST), the wells were blocked by PBS containing 1% skim milk (200 μl/well) for 1 h at 37°C. Then 100 μl of the culture supernatants (1:1,000 in PBS) or diluted MAbs (1:20,000 in PBS) were added to each well and incubated for 1 h at 37°C. Goat anti-mouse IgG (H+L) peroxidase conjugate diluted (1:10,000) was added with 100 μl each well and incubated at 37°C for 1 h. After adding ABTS solution to develop color, the results of the indirect ELISA were measured at 405 nm using a microplate reader. The limit of detection (LOD) was obtained using the method described by Kim *et al*. (2023) [22].

### Antigenicity Changes of Peanut TSSP by Different Heating Methods and Times

In general, roasted peanuts and peanut butter have been widely consumed, while steamed peanuts are also consumed in Asia and Africa [23]. The MAbs developed in this study strongly react to TSSPs in peanuts. Therefore, retention of antigenicity of TSSP by heat treatments including steaming and roasting commonly used in the food industries, is a vital factor in detecting target foods by immunoassays. Peanuts (100 g) were respectively steamed (100°C) and roasted (150 and 200°C) for 0 to 60 min. After cooling at room temperature, proteins were extracted by the heating extraction method described above. Then, 100 uL of the extracts (undiluted) were coated on a 96-well microplate and analyzed by indirect ELISA.

### Detection of Peanut Adulterated in Processed Foods

Peanut is widely used in various popular foods, such as cookies, biscuits, sauces, candy, and chocolate [24]. Thus, we validated whether the developed indirect ELISA and Western blot methods could accurately analyze peanuts adulterated in processed foods. Sixteen types of processed foods (8 cookies, 2 biscuits, 2 sauces, 1 pie, 1 candy, 1 chocolate, and 1 fish jerky) were purchased locally. Among them, 10 food samples (*i.e.*, 4 cookies, 2 sauces, 1 biscuit, 1 candy, 1 chocolate, and 1 fish jerky) were labeled as containing peanuts on their packaging (Table 3). Additionally, processed peanuts (roasted and steamed) and peanut butter were subjected as positive samples for indirect ELISA and Western blot analyses. The results of both methods were compared with those of commercially available kits (ELISA kit 1 [Veratox for Peanut; Neogen, USA] and ELISA kit 2 [AgraQuant Peanut; Romer Labs, USA]). The sample preparation for peanut ELISA kits was carried out following the instructions provided by the manufacturer.

### Statistical Analysis

Each investigation was performed in triplicate and analyzed using SigmaPlot 10.0.1 for Windows (Systat Software, Inc., USA) and Microsoft Excel (Microsoft Corp., USA). The analysis of variance (ANOVA) test, frequency analysis, and descriptive statistics of SPSS 27.0 software (SPSS Inc., IBM, USA) was used to clarify significant differences among groups. A significance level of *p* < 0.05 was used to determine statistical significance. The data were presented as the mean ± standard deviation (SD) from three independent assays (*n* = 3).

## Results and Discussion

### Selection of Conditions and Buffers to Enhance TSSPs Extraction Efficiency

**Fig. 1:**
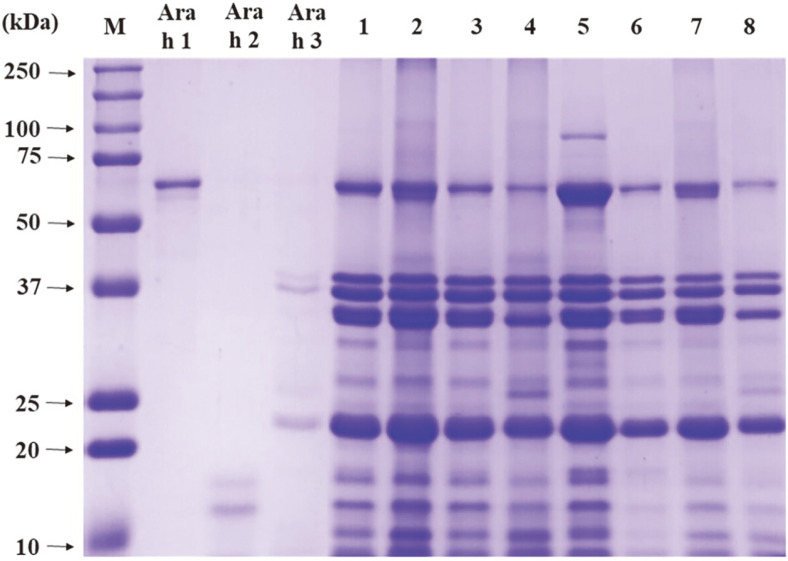
SDS-PAGE patterns of proteins extracted from raw and processed peanuts. M: standard protein marker, Ara h 1, 2, and 3: major peanut allergens, 1: raw (heating), 2: steamed (heating), 2: roasted (heating), 4: peanut butter (heating), 5: raw (non-heating), 6: steamed (non-heating), 7: roasted (non-heating), 8: peanut butter (non-heating).

This study hypothesized that even though the TSSPs in peanut does not induce an allergic reaction, they can be used as a protein marker to identify adulterated peanuts in processed foods heated for a long time [17, 25]. The extracts of peanuts (raw, steamed, and roasted peanut) and peanut butter by heating and non-heating extraction methods were analyzed via the SDS-PAGE and compared with the protein patterns of major allergens Ara h 1, 2, and 3 (Fig. 1). The gel electrophoresis image exhibited similarity of the protein patterns of the extracts obtained by heating and non-heating extraction methods. Additionally, proteins of 64, 17–19, and 14–45 kDa identified in peanut extracts were presumed to be Ara h 1, 2, and 3 [26]. Proteins extracted from raw peanuts using the non-heating extraction method were almost identical to heat-extracted raw, steamed, and roasted peanut extracts. Therefore, it indicated that most proteins in the raw peanut were identified as TSSPs, and their buffer solubility was maintained even after the heat treatment. The protein concentration of peanut extracts by heating and non-heating extractions with 0.1 M carbonate buffer was compared (Table 1). The non-heating treated raw peanut extract exhibited the highest protein concentration (21.0 ± 1.5 mg/ml), whereas the steamed peanut extracted by the same method exhibited the lowest protein concentration (5.8 ± 0.8 mg/ml). However, the protein concentration of raw peanuts was decreased after the heat treatment. This result indicates that some soluble proteins in the raw peanut were denatured and decreased their solubility by heat, but most proteins kept their solubility even after the heat treatment [27].

Moreover, five different buffer conditions were assessed to determine the TSSPs extraction efficiency in this study. The extraction efficiency was compared by measuring the total soluble protein concentration in processed peanuts (steamed and roasted) and peanut butter extracts using heating and non-heating methods (Table S1). The investigation revealed that peanut butter, steamed peanuts, and roasted peanut in carbonate buffer (0.1 M, pH 9.6) showed the highest protein extraction efficiency, followed by 0.05 M PBS in order of efficiency [28]. Additionally, the extracts by heating extraction exhibited higher protein concentration than those from non-heating extraction. The results indicate that the heating extraction method is more effective than the non-heating extraction method in extracting TSSPs from processed peanuts and food containing peanuts [29].

### Characterization of MAbs Specific to Peanut TSSPs

**Fig. 2:**
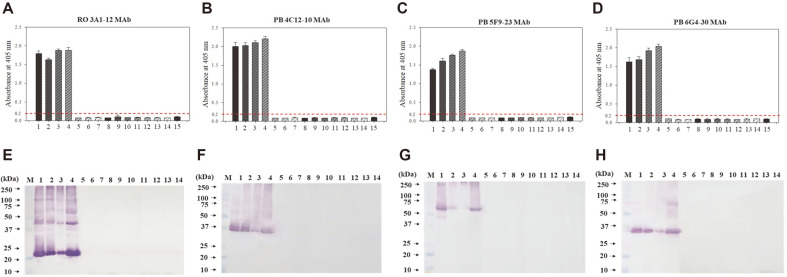
Determination of cross-reactivity for the developed four monoclonal antibodies (RO 3A1-12, PB 4C12-10, PB 5F9-23, and PB 6G4- 30) by indirect ELISA (A−D) and Western blot (E−H). M: standard protein marker, 1: raw peanut, 2: steamed peanut, 3: roasted peanut, 4: peanut butter, 5: almond, 6: cashew nut, 7: soybean, 8: wheat, 9: sesame, 10: shrimp, 11: mackerel, 12: pork meat, 13: egg white 14: milk, 15: negative control for ELISA (0.1 M carbonate buffer, pH 9.6). An absorbance of 0.2 or less was judged to be negative in indirect ELISA. Values represent as mean ± SD (*n* = 3).

**Fig. 3:**
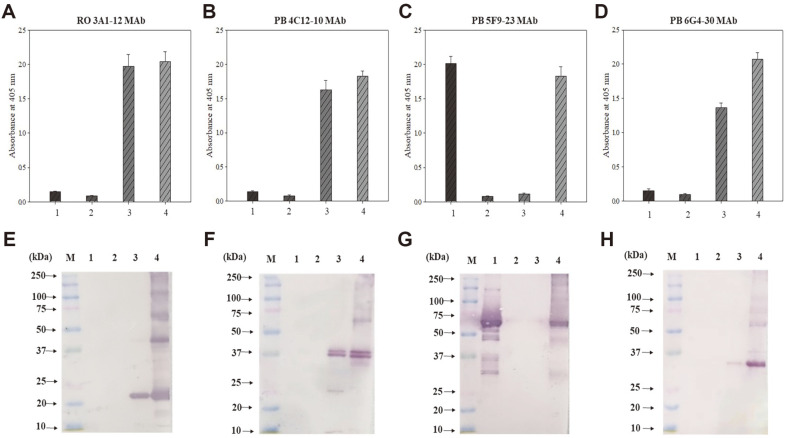
Detection of monoclonal antibodies (RO 3A1-12, PB 4C12-10, PB 5F9-23, and PB 6G4-30) for Ara h 1, 2, and 3 and peanut butter by indirect ELISA (A−D) and Western blot (E−H). M: standard protein marker, 1: Ara h 1 (64.5 kDa, monomer), 2: Ara h 2 (17−19 kDa, doublet), 3: Ara h 3 (14−45 kDa, Multiple subunits), 4: peanut butter. Values represent as mean ± SD (*n* = 3).

Crude extracts containing TSSPs of peanut butter and roasted peanuts were obtained by the heating extraction method and used as immunogens. Mice immunized with the crude extracts showed high titers of high anti-sera, and spleens of mice with high titers were used for cell fusion. Four cloned hybridoma cell lines (RO 3A1-12, PB 4C12-10, PB 5F9-23, and PB 6G4-30) were developed. The 4 MAbs specifically reacted to extracts of raw, steamed, and roasted peanut and peanut butter obtained using the heating extraction method. However, they did not react to the extracts of other food allergens (almond, cashew nut, soybean, wheat, sesame, shrimp, mackerel, pork meat, egg white, and milk) in the indirect ELISA analysis (Fig. 2). In particular, each MAb strongly reacted to proteins of different sizes in Western blot analysis and bound with the same proteins present in raw, steamed, and roasted peanut and peanut butter extracts. Meanwhile, this study also validated the reactivity of the 4 MAbs for the major peanut allergenic proteins Ara h 1, 2, and 3. The crude extract of peanut butter was used as a positive standard. Fig. 3 shows indirect ELISA (Fig. 3A–3D) and Western blot (Figs. 3E–3H) results measuring the interaction between 4 MAbs and Ara h 1, 2, and 3 proteins. The results revealed that PB 5F9-23 MAb and the other 3 MAbs (PB 6G4-30, PB 4C12-10, and RO 3A1-12) reacted to Ara h 1 (64 kDa) and Ara h 3 (14–45 kDa) proteins, respectively. However, the 4 MAbs did not respond to Ara h 2. Chen *et al*. (2019) and Palmer *et al*. (2005) [30, 31] reported that the size of Ara h 1 is 64 kDa, and Ara h 3 is composed of several sizes of proteins. SDS-PAGE results also confirmed proteins with a size of 14–45 kDa (Fig. 1). In Western blot results, three MAbs, such as PB 6G4-30, PB 4C12-10, and RO 3A1-12, reacted to 35 kDa, 37 and 40 kDa, and 22 kDa of Ara h 3, respectively.

We presumed that the low molecular weight (MW) proteins in peanuts were polymerized with a dimer or trimer by heating or that the high MW protein was degraded to the low molecular proteins [32]. Thus, the RO 3A1-12 MAb reacted with different-size proteins at 22, 40, and 60 kDa, and PB 5F9-23 MAb bound to 32 and 64 kDa proteins. Chen and Hsieh (2021) also reported that monoclonal antibodies against TSSP in fish meat reacted simultaneously with proteins of different sizes and presumed that the protein was degraded or polymerized as we mentioned above [33]. Although a further experiment is required, Western blot results revealed that the developed 4 MAbs respond to the allergens Ara h 1 and 3 proteins widely recognized as TSSPs [34]. Therefore, the 4 MAbs developed in this study specifically react to Ara h 1 and 3 in peanuts and could be used as bio-receptors that efficiently detect peanuts in processed foods.

### Sensitivity Enhancement of Indirect ELISA Using an Antibody Cocktail Solution

**Fig. 4:**
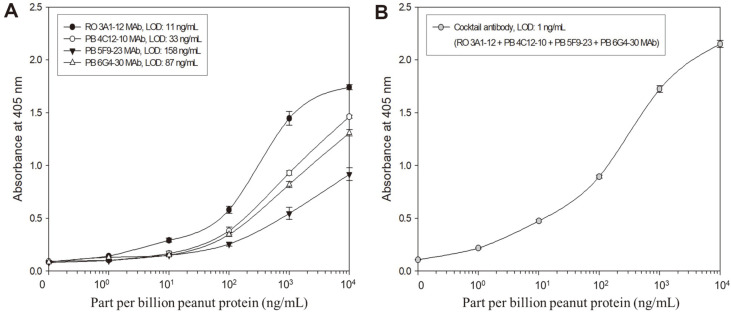
Standard curves of the indirect ELISA method based on the single antibody (A) and antibody cocktail solution (B). The antibody cocktail solution comprises RO 3A1-12, PB 4C12-10, PB 5F9-23, and PB 6G4-30 MAbs at the same concentration used for the single antibody. LOD: Limit of detection. Values represent as mean ± SD (*n* = 3).

The developed MAbs were identified to be specific to peanut TSSPs. Thus, we used all MAbs to develop an indirect ELISA for the sensitive detection of peanuts. As shown in Fig. 4A, among four MAbs, the RO 3A1-12 MAb showed the highest sensitivity (11 ng/ml) in the indirect ELISA. On the hand, we used an antibody cocktail solution (mixture of 4 MAbs at the same concentration) as a primary antibody to enhance the sensitivity Fig. 4B. Through the calculation formula, the LOD of the indirect ELISA based on the antibody cocktail solution was confirmed to be 1 ng/ml. The LOD of the indirect ELISA using the antibody cocktail solution was 11 times enhanced compared to that of a single RO 3A1-12 MAb based-indirect ELISA. Because the MAbs might bind to different epitopes on each peanut TSSP (Fig. 3E–3H), the antibodies (anti-peanut mouse IgG) in the antibody cocktail solution which can be recognized by goat anti-mouse IgG peroxidase conjugate used in ELISA will bind more to the peanut protein coated on the well. The MAbs bound more may be recognized by goat anti-mouse IgG peroxidase conjugate used as a secondary antibody. This leads to an increased sensitivity of the indirect ELISA based on the antibody cocktail solution. Although further research is required, there is an increased sensitivity of indirect ELISA if the antibody cocktail solution simultaneously reacts to numerous proteins derived from food compared to single antibody detection ability.

We also compared the sensitivities and target antigens of the developed indirect ELISA to immunoassays reported in other reports [36-38] (Table 2). The lateral flow immunoassay is specific to native purified Ara h 1, and sandwich ELISA based on a monoclonal antibody specific to recombinant Ara h 2 of peanuts has been reported previously. They can detect peanuts in samples with a trace amount of 10 and 5 ng/ml, respectively. However, the indirect ELISA used in this study exhibited higher sensitivity (LOD 1 ng/ml) and was bound to 14–45 and 60, and 64 kDa TSSPs, which are assumed to be peanut allergens Ara h 1 and 3.

### Antigenicity Changes of Peanut TSSP in Different Heating Methods and Times

Peanut has been consumed in versatility in processing all over the world [35]. Heat treatment is the most representative method used in food industries to improve the shelf life and flavor of peanuts. It is well known that most proteins in food were turned to insoluble status and lost their antigenicity due to denaturation during the heat treatment, but some proteins have been reported to be able to maintain solubility and antigenicity [33].

**Fig. 5:**
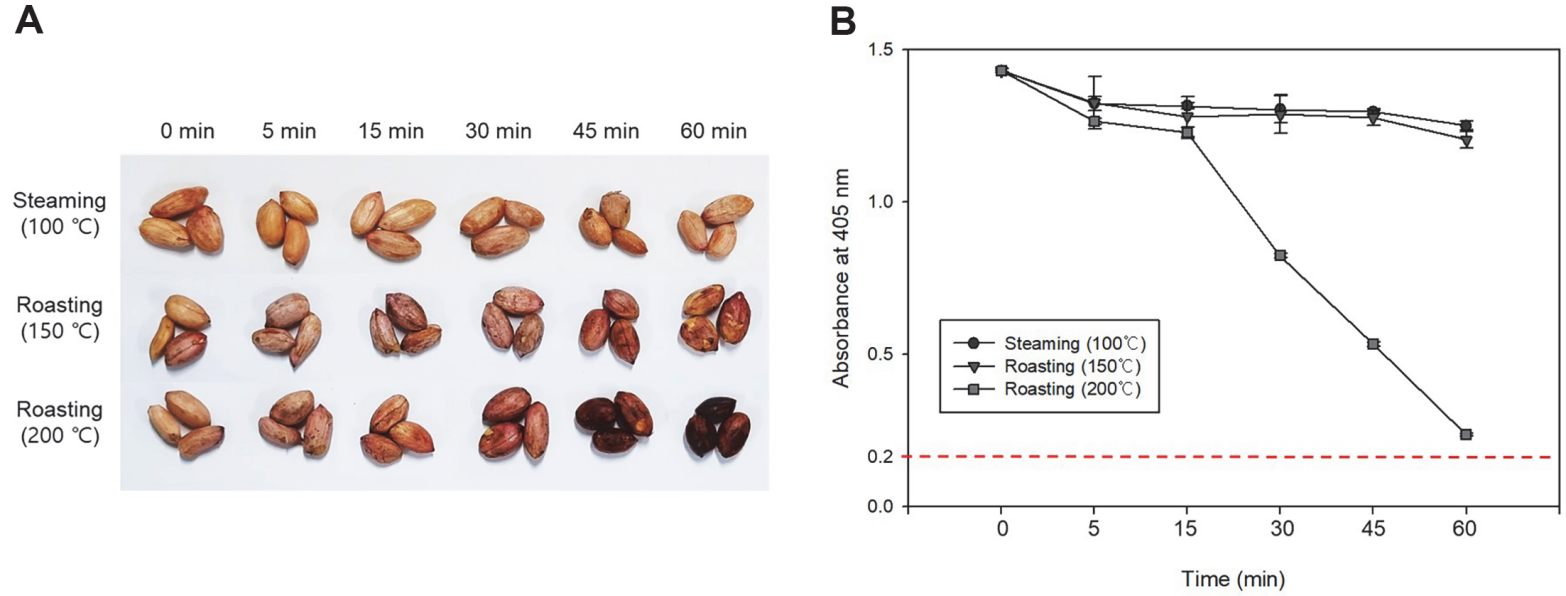
Antigenicity changes of peanut TSSP in different heating methods. (**A**) The image shows the status of the peanut after heat treatment. (**B**) Liner graphs are the analytical results of the indirect ELISA based on the antibody cocktail solution. An absorbance of 0.2 or less was judged to be negative in indirect ELISA. Values represent as mean ± SD (*n* = 3).

In the indirect ELISA analysis, a sample showing more than 0.2 OD value (OD value of 0% peanut sample + 5 × SD of the OD value of 0% peanut sample) was classified to be a positive sample [22]. As shown in Fig. 5, the extracts from steamed and mild roasted peanuts showed an average of 1.3 OD values in the indirect ELISA analysis. This meant that TSSPs kept their antigenicity even after steaming (100°C for 60 min) and mild roasting (150°C for 60 min). Meanwhile, when peanut was roasted at 200°C for 15 min, TSSP antigenicity was maintained but decreased after 30 min. The peanut treated for more than 30 min at 200°C started to burn and was not edible status.

This result proves that the TSSPs in peanuts as target proteins can retain their antigenicity after general heat treatments used in food industries to produce edible foods. It can be a useful biomarker for the indirect ELISA to classify peanuts adulterated in processed foods rapidly.

### Detection of Peanut Adulterated in Processed Foods by the Developed Indirect ELISA and Commercialized ELISA Kits

Sixteen processed food samples were collected to evaluate whether the indirect ELISA based on the antibody cocktail solution can detect peanuts in foods. Steamed and roasted peanuts and peanut butter were used as positive controls. As we mentioned above, a sample exhibiting an absorbance of 0.2 or more in the indirect ELISA was judged to be a positive sample (Fig. S1 and Table 3). The results of the indirect ELISA indicated that 3 types of peanuts (steamed, roasted, and peanut butter) and 10 food samples with peanuts were classified as positive. Conversely, 6 food samples without peanuts were all negative. The results of antibody cocktail solution based-indirect ELISA and -Western blot for the peanut detection in processed food samples showed reliable arrangement with those of commercially available peanut ELISA Kits. This result indicates that the indirect ELISA, based on the antibody cocktail solution, is a reliable and effective tool for analyzing peanut adulteration in processed foods. Furthermore, the results obtained in this study demonstrate that the TSSPs used as the target protein in peanuts retain their antigenicity even after numerous thermal processing methods. These TSSPs can be successfully detected by the developed MAbs.

## Conclusion

Peanuts are well-known as food allergens, so it is necessary to quickly determine whether they exist in food processing facilities or are adulterated into processed foods, especially when products do not label peanuts as an ingredient. This study confirmed that the TSSPs present in peanuts still maintain heat stability and buffer solubility even after long-term heat treatment. In particular, heating at boiling water for 15 min is necessary to enhance the extraction efficiency of TSSPs from processed foods. TSSP pattern in peanut butter and peanuts (raw, roasted, and steamed) was the same in the SDS-PAGE analysis. The results meant TSSPs in raw peanuts could maintain water solubility without being denatured to insolubility even when the peanut had been heated for a long time.

Given the higher sensitivity and specificity, RO 3A1-12 MAb was chosen for optimizing indirect ELISA and Western blot analyses. The indirect ELISA based on RO 3A1-12 MAb detected peanuts in processed foods. In addition, an antibody cocktail solution was used to enhance the sensitivity (1 ng/ml) of indirect ELISA, and antibody cocktail solution based-indirect ELISA successfully detected peanuts in processed foods. This study also demonstrated that the TSSPs present in peanut was not denatured even after the heat treatment used in general food manufacturing processes. Nonetheless, all in vitro results indicated that TSSPs could be used as a valuable biomarker to determine whether peanuts are adulterated into processed foods. The developed MAbs in this study are expected to be used to develop various biosensors based on antibodies specific to TSSPs in peanuts.

## Supplemental Materials

Supplementary data for this paper are available on-line only at http://jmb.or.kr.

## Figures and Tables

**Table 1:** Quantification of peanut proteins extracted by heating and non-heating extraction methods.

Samples	Protein concentration (mg/ml)	
	Non-heating extraction	Heating extraction
Raw peanut	21.0 ± 1.5	14.8 ± 0.1
Steamed peanut	5.8 ± 0.8^cd^	9.9 ± 0.03^ac^
Roasted peanut	8.1 ± 1.6^bc^	12.7 ± 1.4^ab^
Peanut butter	9.6 ± 2.7^bd^	19.2 ± 3.8

The difference in protein concentration between each sample (peanut butter, roasted peanut, steamed peanut) and extraction method was statistically significant (*p* < 0.01), values followed by different superscript letters are significantly different (*p* < 0.05) by one-way ANOVA with Tukey test.

**Table 2:** Comparison of the method developed with other immunoassays previously reported for peanut protein.

Detection techniques	Target antigen	Limits of detection	References
Lateral flow immunoassay	Native purified peanut Ara h 1 protein	10 ng/ml	[36]
Sandwich ELISA	Recombinant Ara h 2	5 ng/ml	[37]
Competitive direct ELISA	Ara h 1 Ara h 2	190 ng/ml 60 ng/ml	[38]
Indirect ELISA	Peanut thermal stable-soluble proteins	1 ng/ml	This study

**Table 3:** Comparative analysis for food samples by antibody cocktail solution (RO 3A1-12, PB 4C12-10, PB 5F9-23, and PB 6G4-30) based Western blot and indirect ELISA and commercially available ELISA kits.

Food sample	Peanuts content	Methods used in this study		ELISA kits	
		Western blot	ELISA	1	2
Steamed	○^a^	+^b^	+	+	+
Roasted	○	+	+	+	+
Peanut butter	○	+	+	+	+
Biscuit 1	○	+	+	+	+
Cookie 1	○	+	+	+	+
Cookie 2	○	+	+	+	+
Cookie 3	○	+	+	+	+
Fish jerky	○	+	+	+	+
Sauce 1	○	+	+	+	+
Sauce 2	○	+	+	+	+
Chocolate	○	+	+	+	+
Candy	○	+	+	+	+
Cookie 4	○	+	+	+	+
Cookie 5	×^c^	-^d^	-	-	-
Cookie 6	×	-	-	-	-
Biscuit 2	×	-	-	-	-
Pie	×	-	-	-	-
Cookie 7	×	-	-	-	-
Cookie 8	×	-	-	-	-

a,b,c,dRepresentation of symbols: ○, a food contains peanuts; ×, a food does not contain peanuts; +, positive (≥ LOD); -, negative (<LOD). ELISA kit 1: Veratox for Peanut (Neogen); LOD: 1 ppm peanut. ELISA kit 2: AgraQuant Peanut (Romer Labs); LOD: 0.1 ppm peanut.
