# Supplementary material for: Detection of a Thermal Stable-Soluble Protein (TSSP) as a Marker of Peanut Adulteration Using a Highly Sensitive Indirect Enzyme-Linked Immunosorbent Assay based on Monoclonal Antibodies
Source: J Microbiol Biotechnol. 2023 May 30;33(9):1170–8. doi: 10.4014/jmb.2304.04038 (PMC10580888; doi:10.4014/jmb.2304.04038)
Supplement: Supplementary file 1 [file jmb-33-9-1170-supple.pdf]

https://doi.org/10.4014/jmb.2304.04038

Supplementary Figure and Table

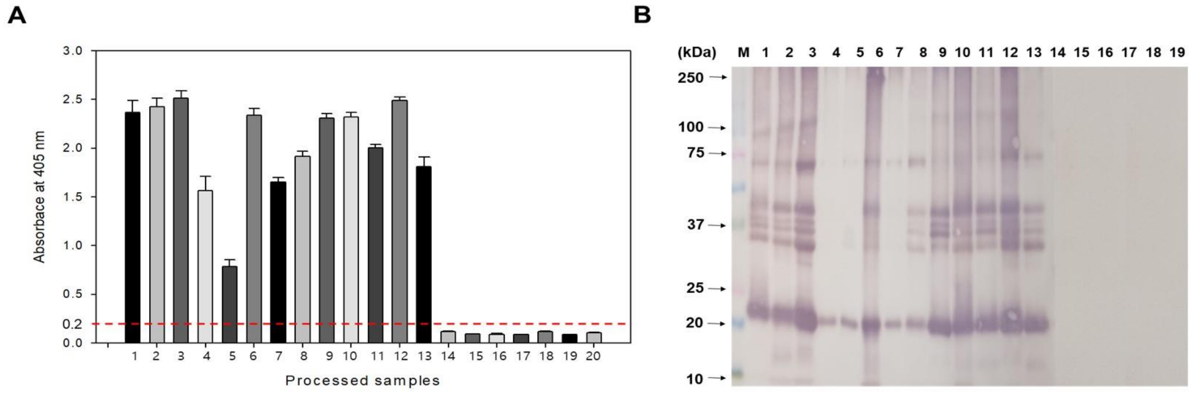

Fig. S1. Analytical results of processed foods by the indirect ELISA **(A)** and Western blot **(B)** based on the 4 MAbs (RO 3A1-12, PB 4C12-10, PB 5F9-23, and PB 6G4-30). M: marker, 1: steamed peanut, 2: roasted peanut, 3: peanut butter, 4: biscuit 1, 5: cookie 1, 6: cookie 2, 7: cookie 3, 8: fish jerky, 9: sauce 1, 10: sauce 2, 11: chocolate, 12: candy, 13: cookie 4, 14: cookie 5, 15: cookie 6, 16: biscuit 2, 17: pie, 18: cookie 7, 19: cookie 8, 20: negative control for ELISA (0.1 M carbonate buffer, pH 9.6). An absorbance of 0.2 or less was judged to be negative in indirect ELISA. Values represent as mean ± SD (*n* = 3).

Table S1. Protein concentration of peanut butter, roasted peanut, and steamed peanut extracts.

(protein concentration: mg/mL)

Sample(s)	Extract method(s)	Extraction buffer(s)				
		0.05 M PBS (pH 7.4)	0.1 M Carbonate (pH 9.6)	0.5 M NaCl (pH 6.5)	0.025 M TBS (pH 7.4)	0.02 M Tris-HCl (pH 7.4)
Peanut butter	Non-heating	6.0 ± 1.5^{b}	9.6 ± 2.7^{a}	1.5 ± 0.8^{de}	0.7 ± 0.3^{ce}	1.3 ± 0.5^{cd}
	Heating	12.5 ± 2.4	19.2 ± 3.8	2.9 ± 1.1^{de}	1.5 ± 0.2^{ce}	0.8 ± 0.3^{cd}
Roasted peanut	Non-heating	2.6 ± 1.1^{cde}	8.1 ± 1.6	0.7 ± 0.4^{a}	0.5 ± 0.3^{ace}	1.8 ± 0.1^{acd}
	Heating	4.3 ± 1.2^{ce}	12.7 ± 1.4	2.1 ± 0.9^{de}	1.4 ± 0.7^{ce}	1.9 ± 0.4^{cd}
Steamed peanut	Non-heating	4.6 ± 0.3^{be}	5.8 ± 0.8^{a}	2.7 ± 0.1^{e}	0.9 ± 0.1^{a}	3.8 ± 0.5^{ac}
	Heating	6.3 ± 0.4^{e}	9.9 ± 0.4	3.0 ± 0.3^{d}	1.5 ± 0.1^{c}	4.9 ± 1.1^{a}

Non-heat: kept on ice for 1 h, heat: placed in boiling water for 15 min. The concentration difference between each sample (peanut butter, roasted peanut, steamed peanut) and extraction buffer was statistically significant ($p<0.001$), values followed by different superscript letters are significantly different ($p<0.05$) by one-way ANOVA with Tukey test.
